# Left Ventricular Diastolic Function Studied with Magnetic Resonance Imaging: A Systematic Review of Techniques and Relation to Established Measures of Diastolic Function

**DOI:** 10.3390/diagnostics11071282

**Published:** 2021-07-16

**Authors:** Annemie Stege Bojer, Martin Heyn Soerensen, Peter Gaede, Saul Myerson, Per Lav Madsen

**Affiliations:** 1 Department of Cardiology and Endocrinology, Slagelse Hospital, 4200 Slagelse, Denmark; mahso@regionsjaelland.dk (M.H.S.); phgo@regionsjaelland.dk (P.G.); 2 Institute of Regional Health Research, University of Sothern Denmark, 5230 Odense, Denmark; 3 Centre for Clinical Magnetic Resonance Research, University of Oxford, Oxford OX1 2JD, UK; saul.myerson@cardiov.ox.ac.uk; 4 Department of Cardiology, Copenhagen University Hospital, 2730 Herlev, Denmark; lav.madsen@gmail.com; 5 Department of Clinical Medicine, University of Copenhagen, 2200 Copenhagen, Denmark

**Keywords:** cardiovascular magnetic resonance, diastology, tagging, left ventricle time-volume curve, peak filling rate, feature tracking, left atrium time-volume curves, velocity-encoded phase-contrast

## Abstract

**Purpose:** In recent years, cardiac magnetic resonance (CMR) has been used to assess LV diastolic function. In this systematic review, studies were identified where CMR parameters had been evaluated in healthy and/or patient groups with proven diastolic dysfunction or known to develop heart failure with preserved ejection fraction. We aimed at describing the parameters most often used, thresholds where possible, and correlation to echocardiographic and invasive measurements. **Methods and results:** A systematic literature review was performed using the databases of PubMed, Embase, and Cochrane. In total, 3808 articles were screened, and 102 studies were included. Four main CMR techniques were identified: tagging; time/volume curves; mitral inflow quantification with velocity-encoded phase-contrast sequences; and feature tracking. Techniques were described and estimates were presented in tables. From published studies, peak change of torsion shear angle versus volume changes in early diastole (−dφ′/dV′) (from tagging analysis), early peak filling rate indexed to LV end-diastolic volume <2.1 s^−1^ (from LV time-volume curve analysis), enlarged LA maximal volume >52 mL/m^2^, lowered LA total (<40%), and lowered LA passive emptying fractions (<16%) seem to be reliable measures of LV diastolic dysfunction. Feature tracking, especially of the atrium, shows promise but is still a novel technique. **Conclusion:** CMR techniques of LV untwisting and early filling and LA measures of poor emptying are promising for the diagnosis of LV filling impairment, but further research in long-term follow-up studies is needed to assess the ability for the parameters to predict patient related outcomes.

## 1. Introduction

Impaired diastolic filling of the heart is a well-recognized cause of heart failure (HF), but it has proven difficult to measure and diagnose accurately. A reference standard is the finding of invasively measured increased mid- or end-diastolic (EDP) left ventricular (LV) filling pressure, but an invasive measurement in all patients has never been a realistic option. Furthermore, it is desirable to identify patients in earlier stages of impaired diastolic heart function before LV diastolic pressures are elevated, and thus while it may still be possible to reverse ventricular remodeling. Most studies on LV diastolic dysfunction have relied on echo- and tissue-Doppler interrogation of mitral valve diastolic inflow patterns and relaxation of LV myocardium. Echo-Doppler is versatile, and a high number of echo-determined parameters have been related to different aspects of diastolic (dys-)function, but there is overlap with normal values, and cut-off values for diastolic dysfunction have been difficult to establish. Partly due to this difficulty in assessing diastolic function, the term “HF with preserved ejection fraction” (HFpEF) was coined in the 1990’s. However, the term has not been altogether helpful, as it may result in a pooling of very different patient populations [1,2], which may partly explain the negative outcome trials [3,4,5]. The 2016 ASE/EACVI guidelines [6] simplified and sharpened criteria for diastolic dysfunction, and recent studies [7,8] comparing echo-parameters with invasively determined LVEDP have found that the new guidelines raised specificity and lowered sensitivity in comparison with earlier echo criteria. This is helpful in the establishment of frank LV diastolic dysfunction as a cause of dyspnoea but still does not take into consideration that the mechanism leading to increased pressures can be very different between patients. Hence, interventions aiming at improving early LV untwisting (ATP-dependent) may not be the same as interventions aiming at lessening LV myocardial fibrosis deposition.

Cardiovascular magnetic resonance (CMR) has not traditionally been considered a technique to evaluate diastolic dysfunction but may have an important role in acquiring accurate measures due to its high spatial resolution. Thus, CMR is a reference technique for three-dimensional coverage of volumes of the LV and left atrium (LA) in addition to the evaluation of myocardial composition, mass, and function. Previous reviews of CMR assessment of diastolic dysfunction, including expert opinions and state-of-the-art papers [9,10,11], have examined different aspects of LV diastole. Nevertheless, diastology with CMR is still a confusing field with little consensus leading to the use of a variety of different CMR techniques and parameters, with studies spanning more than three decades. In this work, we attempt to present a broad overview of the field with a systematic review of current clinical CMR studies where diastolic dysfunction has been analyzed. We have aimed at identifying studies with CMR parameters related to invasively determined LV diastolic pressures or to 2016 echo-Doppler ASE/EACVI criteria [6], but for the review to be able to stimulate and guide further academic thinking, we have also included studies on diastolic function in healthy young, in elderly people, and in patients with diseases known to have a high propensity to develop diastolic dysfunction and studies assessing correlation to adverse clinical outcomes. Using this two-legged approach, we aim to provide an overview of the most studied current CMR techniques. It has been our aim to provide normal values for the obtained parameters, to provide guidance for values in specific patient groups that are known to develop diastolic heart failure, and specifically to identify the parameters currently best evaluated against reference standards (echo-Doppler and/or invasive studies).

## 2. Materials and Methods

### 2.1. Systematic Search and Eligible Studies

The PubMed.gov (National Library of Medicine, USA), EMBASE.com (Elsevier, Amsterdam, The Netherlands), and Cochrane.org (Cochrane Collaboration, London, UK) databases were searched in June 2021. The following terms were used in the systematic search strategy: “CMR” OR “cardiovascular magnetic resonance” OR “magnetic resonance” OR “MRI” OR “magnetic resonance imaging, cine” OR “magnetic resonance imaging” AND “peak diastolic strain rate” OR “diastolic function” OR “myocardial tagging” OR “heart failure with preserved ejection fraction” OR “HFpEF” OR “atrial function” OR “left atrial size” OR “LA size” OR “left atrial volume” OR “LA passive emptying fraction” OR “left atrial passive emptying fraction” OR “left ventricle peak filling rate” OR “LV Peak filling rate” OR “LA volumes” OR “filling pressures” OR “transmitral flow” OR “diastolic dysfunction” OR “diastolic filling” OR “heart failure, diastolic” OR “diastole” OR “atrial function”. The search was fitted to the three databases individually.

We included original studies where CMR had been used to evaluate global LV diastolic function in human adults using 1.5T or 3.0T magnetic resonance systems. We excluded studies on patients with LV ejection fraction (EF) <45%, studies on regional diastolic function, and studies on patients with valve disease other than isolated aortic valve stenosis (AS) where diastolic dysfunction becomes important. CMR studies of indirect aspects linked to diastolic dysfunction (myocardial mass and localized/diffuse myocardial fibrosis) were excluded since these have been extensively reviewed elsewhere and may not necessarily indicate diastolic dysfunction in all cases. Case reports, conference abstracts, descriptive studies where no values were presented, non-English publications, pediatric or pregnant patient groups, congenital heart disease, and studies focusing on atrial function and not ventricular function, including patients with atrial fibrillation, were excluded. The older T_1_ cine sequences (i.e., FLASH sequences) produce slightly lower LV volumes than the currently applied steady-state free precession cine images and were excluded. Reference lists of previous systematic reviews were screened for original studies and studies not identified by the initial database search. The protocol has been published on PROSPERO (CRD 42016051982) (crd.york.ac.uk, University of York, York, UK).

After the initial search, duplicates were removed, and remaining articles were screened by title and abstract. We reviewed the full text of all potentially eligible articles. To provide normal reference values, we included studies on healthy young controls and studies on healthy elderly subjects (for age-related LV stiffness), as well as patients with hypertension, diabetes, ischemic heart disease, isolated significant AS, and diagnosed conditions often associated with increased LV mass/LV stiffness: hypertrophic cardiomyopathy (HCM), cardiac sarcoidosis, and cardiac amyloidosis. Echo-Doppler and/or invasive measurements of diastolic function were included for comparison with CMR. Reported echo-Doppler parameters were compared to 2016 American Society of Echocardiography and The European Association of Cardiovascular Imaging criteria where possible [1].

### 2.2. Data Collection

We aimed to provide normal ranges (mean with standard deviation) and differences between normal subjects and patient groups where adequate statistical analysis had been provided (parametric or non-parametric as considered appropriate by the authors) with a two-sided level of <0.05. If more than one study was found, and if estimates were similar, we have presented data from the larger cohort. If deviating significantly, all estimates have been presented.

## 3. Results and Discussion

**Figure 1:**
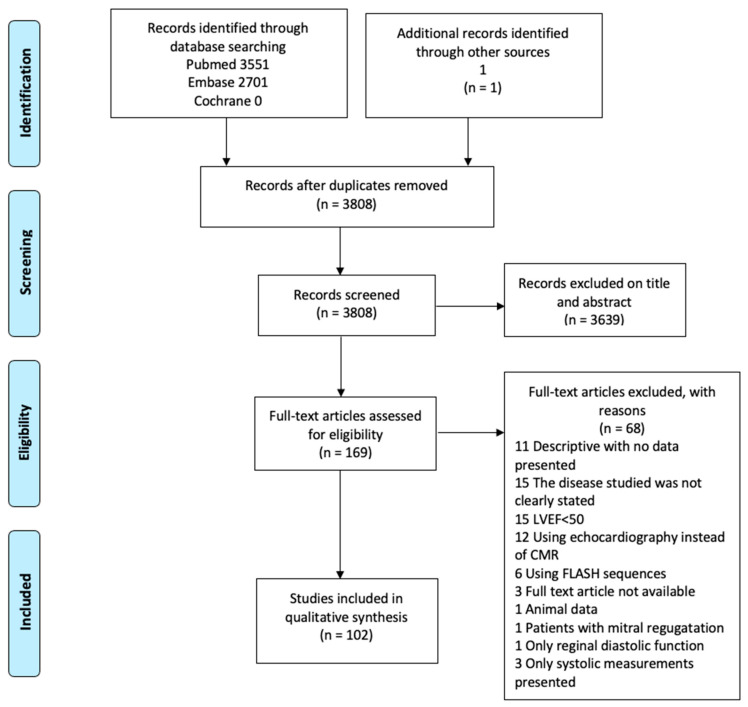
Consort diagram.

**Figure 2:**
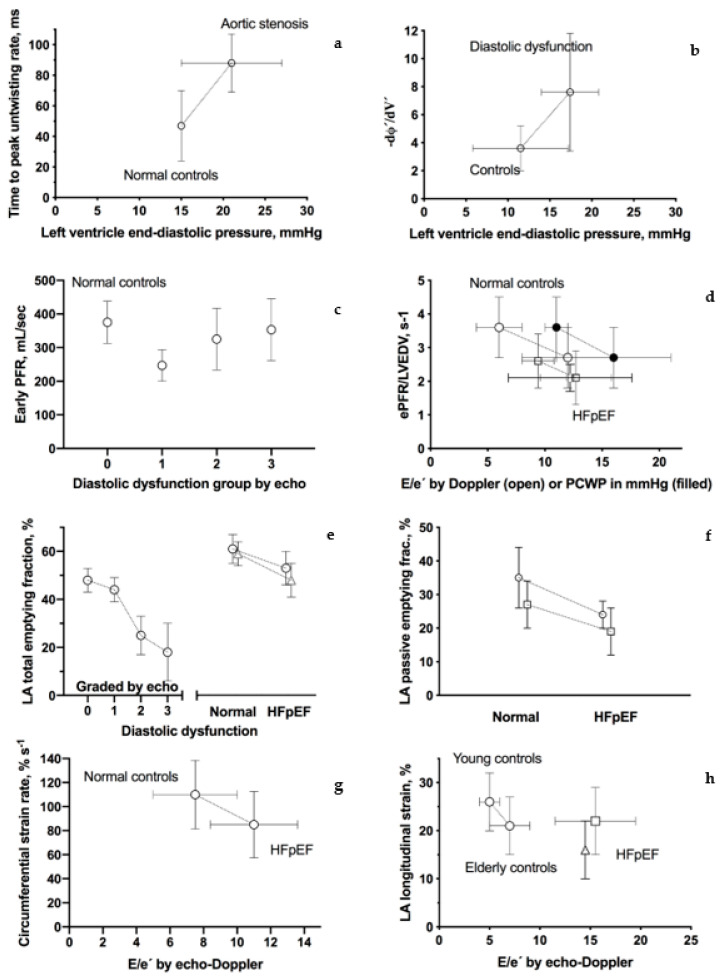
CMR tagging—brief description. Graphic presentation of CMR-assessed parameters of diastolic dysfunction. Connecting lines indicate that estimates come from the same study. References are from the left top corner; (**a**) time to peak untwisting rate (ms) [12]; (**b**) peak change of the torsion shear angle versus volume curve in the early diastole (−dφ′/dV′) [13]; (**c**) early peak filling rate (Early PFR, mL/sec) [14]; (**d**) early peak filling rate indexed to LV end-diastolic volume (ePEF/LVEDV, ms) [15,16] and [17] (only patients with reduced diastolic function, please see text); (**e**) left atrium total emptying fraction (%) [14,18,19]; (**f**) left atrium passive emptying fraction (%) [18,19]; (**g**) circumferential-myocardial strain rate from feature tracking (% s^−1^) [20]; and (**h**) left atrium total longitudinal strain from feature tracking (%) [18,21,22].

As demonstrated in the Consort diagram (Figure 1), 102 scientific papers were included in this review. During the initial literary search, four main techniques were identified: Tagging; LV and LA time/volume curves; mitral inflow quantification with velocity-encoded phase-contrast sequence; and LV and LA feature tracking. In the following, we aim to present each technique followed by results from normal subjects, including studies on intra- and interrater variability, and from studies on patients with suspected or proven diastolic dysfunction. Each subsection ends with a small discussion of the technique, including its drawbacks, but in general, we have emphasized studies where adequate control has been provided by echo-Doppler or invasive means. Thus, results from such studies are presented graphically in Figure 2.

### 3.1. CMR Tagging—Brief Description

**Figure 3:**
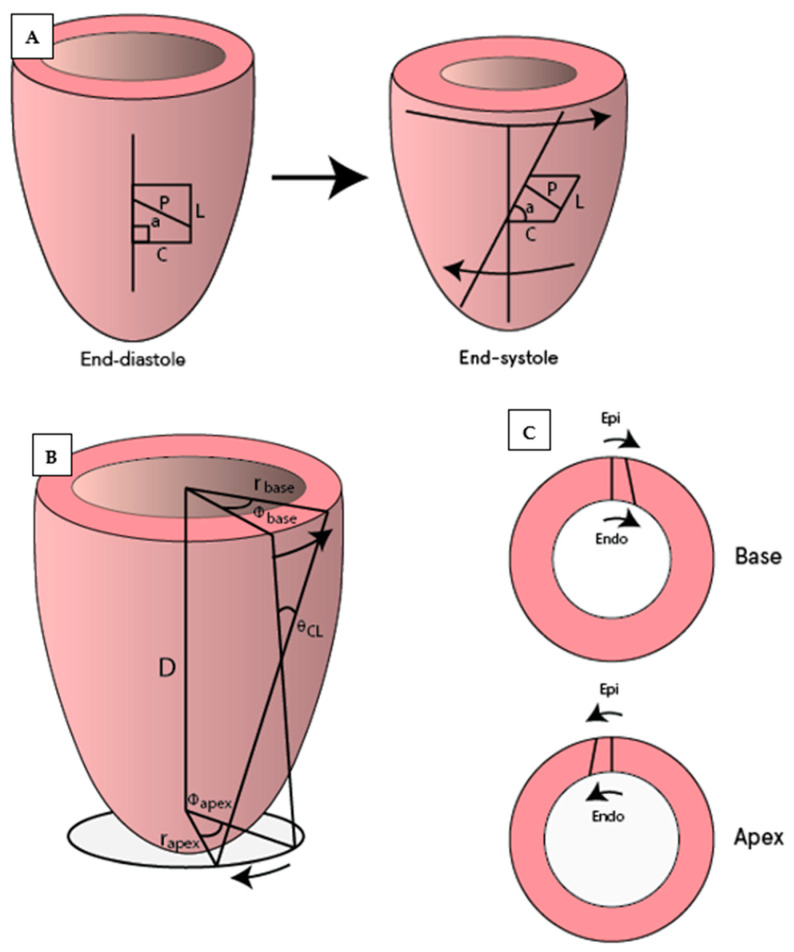
Illustration of commonly used CMR tagging measurements. (**A**) Circumferential (**C**) and longitudinal strain (L), which also allows for measurements of strain rates and shear strain (difference in strain between two locations such as circumferential-longitudinal or circumferential-radial). Measurement of diastolic rotation (a) and untwisting (difference in rotation between defined basal and apical slices; in degrees) and their corresponding rates (temporal changes). (**B**) The torsion shear angle $\theta_{CL}=\frac{\left(\Phi_{\mathrm{apex}}-\Phi_{\mathrm{base}}\right)\left(r_{apex}+r_{base}\right)}{2D}\;$, the change in angle of longitudinal tag lines from the base to the apex on the surface of the heart [23]. (**C**) Circumferential-radial strain.

Saturation bands of magnetization lines are placed across the myocardium to “tag” the desired region creating a visible grid or series of parallel lines on the myocardium. Taglines are tracked during diastole to determine the changes in the distance and twisting between them. With SPAMM sequences (spatial modulation of magnetization) taglines fade during end-diastole, and hence this technique is mainly used to assess LV early diastolic “untwisting” of the LV. With CSAPMM sequences (complementary spatial modulation of magnetization), taglines last throughout the whole of diastole. Corresponding to the relaxation of the LV in the isovolumetric phase of diastole, the basal LV myocardium twists counter-clockwise (denoted with positive degrees) as seen from the apex, while the apex twists clockwise (negative degrees) [23]. Various parameters can be assessed with tagging, the most common of which are illustrated in Figure 3. Fourteen studies applying CMR tagging were identified (Table 1, Table 2, Table 3 and Table 4).

### 3.2. CMR Tagging—Diastolic Rotation/Untwisting

Four studies (*n* = 9–32) reported rotation/untwisting with corresponding rates in healthy young [25,27,28,34] (Table 1). Regional (apical, mid-ventricular, and basal) differences were found, but when absolute values were normalized to peak systolic rotation for the slice in question, they were similar [25]. Thus, using a normalized parameter allows for robust comparisons independent of slice location and degree of systolic twist. Intra- and inter-scan repeatability of the normalized global untwisting rate had a low scan bias of 3.3°/s (−17.3, 10.5°/s) and 2.0°/s (−25.2, 18.1°/s) when assessed in healthy subjects [35]. In comparison with the young, the normalized apical diastolic rotation rate is slightly reduced in the elderly (Table 1) [24], corresponding to impaired relaxation as evidenced by a correspondingly impaired echo-Doppler mitral inflow pattern. In AS patients, the apical rotation rate normalized to peak systolic rotation was lowered by ~36% (Table 1) [26], but AS patients were older than the control group; therefore, the findings could simply be age-related.

### 3.3. CMR Tagging—Diastolic Strain Rates

The diastolic global circumferential-longitudinal shear strain rate has been reported in healthy subjects (Table 2) [25], and five studies have reported on strain rates in different patient cohorts. Unfortunately, no studies describing normal values for healthy patients in different age groups exist. Patients with DM2 or arterial hypertension have a 20–35% reduction in global (average of basal, mid-ventricular, and apical slice) diastolic peak circumferential strain rate and diastolic longitudinal strain rate. The control groups were slightly younger than patient groups, but this was probably not a difference that could explain the effect seen (Table 2) [29,32]. In patients with moderate-to-severe AS, the global diastolic peak circumferential strain rate closely resembled estimates for patients with DM2 and arterial hypertension (Table 2) [30]. One study was found on inter-observer and inter-study reproducibility of global diastolic peak circumferential strain rate in patients with moderate-to-severe AS for both 1.5T and 3T [31]: the inter-observer variability was good for both 1.5T-CSAPMM (coefficient of variation 4%) and 3T-SPAMM (6%), but the inter-study reproducibility was only good-to-moderate for 1.5T-CSAPMM (coefficient of variation 19%) and poor for 3T-SPAMM (34%). In a study of patients with severe AS undergoing transcatheter aortic valve implantation, a moderate correlation (r^2^ = 0.5) exists between decreasing mid-ventricular circumferential diastolic peak strain rate and increasing LVEDP (Table 2) [33]. Unfortunately, estimates for mean LVEDP were not reported, but the results suggest that the mid-ventricular circumferential diastolic peak strain rate decreases with worsening diastolic function.

### 3.4. CMR Tagging—Time to Peak Untwist

Two studies examined time to LV untwist of AS patients and found early diastole to be almost doubled in duration compared to controls (Table 3) [12,26]. In AS patients, the untwisting phase and the filling phase of diastole appeared to overlap, contrasting with findings in normal hearts where untwisting is accomplished before the mitral valve opens. Two further studies reported on time to peak untwist but without stating the starting point for this measurement [25,32].

### 3.5. CMR Tagging—Peak Change of Torsion Shear Angle versus Volume Changes in Early Diastole (−dφ′/dV′)

Two studies reported on the peak early diastolic change of the torsion shear angle versus volume changes (normalized values) (−d**φ**′/dV′). In arterial hypertension, −d**φ**′/dV′ was ~2 times higher than in controls (Table 4) [32], and in patients undergoing coronary angiography for chest pain without acute myocardial infarction, an ~2 times higher -dφ′/dV′ was also found. The −d**φ**′/dV′ is only poorly correlated to LVEDP and Tau (r^2^ = 0.36 and r^2^ = 0.37, respectively) [13], but with a −d**φ**′/dV′ ≥ 6.2, the sensitivity is 72% with a specificity of 100% for identification of LVEDP > 12 mm Hg and Tau > 48 ms.

### 3.6. CMR Tagging—Summarized

Several parameters can be assessed (Table 1, Table 2, Table 3 and Table 4), but no consensus exists on which parameters to use, and more studies are needed for robust normal ranges. When reporting rotation, untwisting, or strain rates, these should be normalized to the peak systolic values, making them independent of slice location and systolic twist. Two studies presented invasive measurements. One study demonstrated an only moderate correlation between decreasing mid-ventricular circumferential diastolic peak strain rate and increasing LVEDP. The other study found that for −dφ′/dV′ ≥ 6.2, both specificity and sensitivity were high for severely compromised diastolic filling, but 95% CI for LVEDP for the two groups overlapped. A further three studies presented echo-Doppler measurements, but in none of these was it possible to assess the diastolic function according to the 2016 ASE/EACVI criteria. Furthermore, one study on inter-study reproducibility of global diastolic peak circumferential strain rate showed moderate-to-poor results for both 1.5T and 3T. Thus, in most studies with conditions related to poor LV unwinding, the tagging measures of diastolic function were reduced by 30–40%, but the technique needs further validation before it can be applied to clinical practice. Additionally, the sequences used are still not analyzable in most standard CMR analysis software, limiting the applicability in a clinical setting.

### 3.7. LV and LA Time/Volume Curves—Brief Description

**Figure 4:**
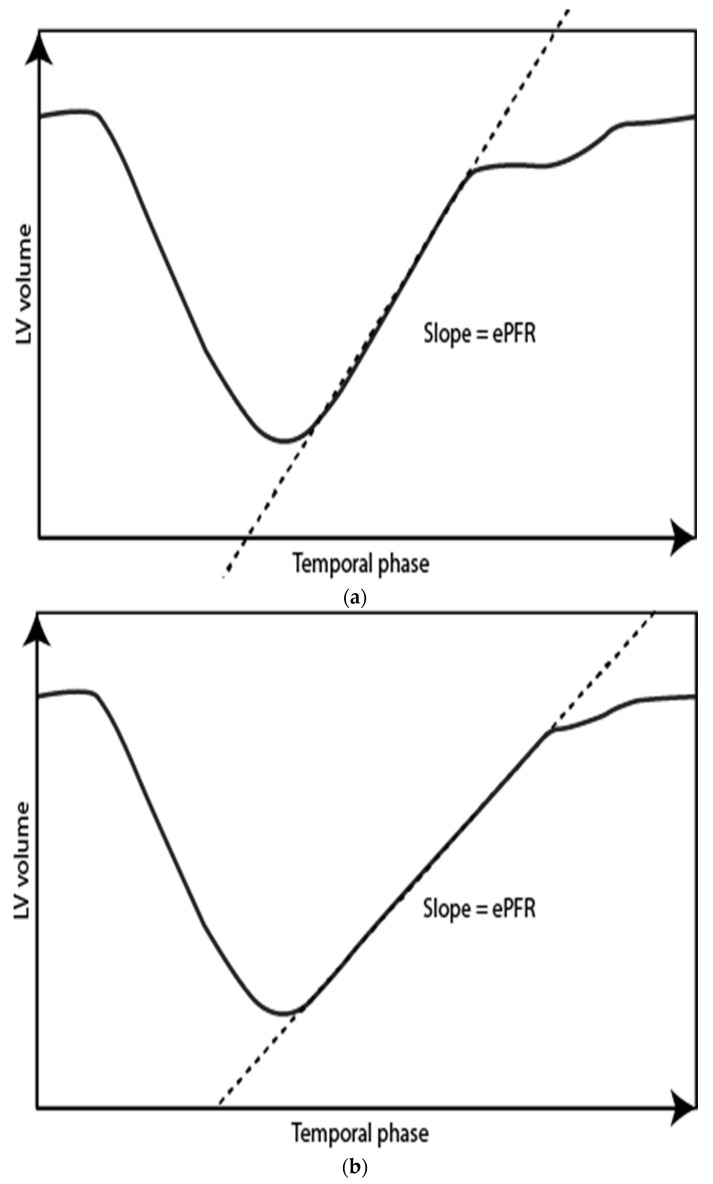
Left ventricle time-volume curves for a normal subject (**a**) and a patient with diastolic impairment (**b**). Notice a more gradual slope in the patient with diastolic impairment corresponding to a lower early peak filling rate = ePFR.

**Figure 5:**
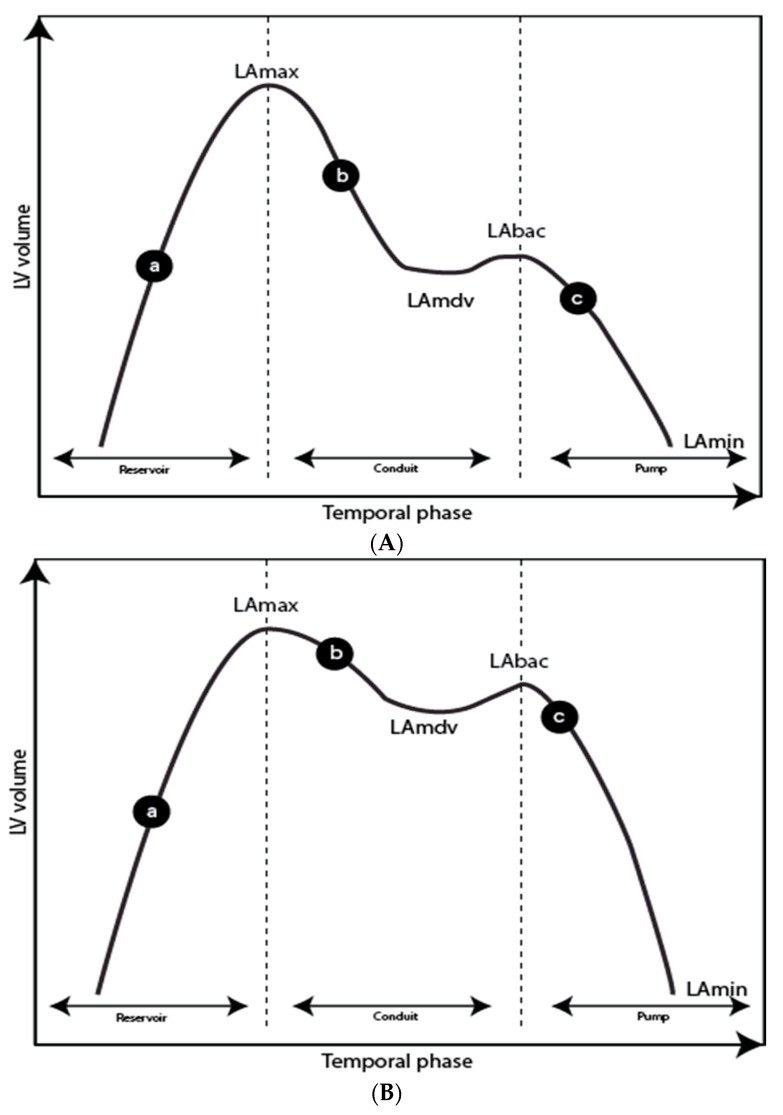
Left atrial (LA) time-volume curves for a normal subject (**A**) and a patient with diastolic impairment (**B**). (a) LA filling during left ventricular systole, (b) LA passive emptying, (c) LA contraction. LA_max_, left atrial maximum volume; LA_mdv_, left atrium mid-diastolic volume and plateau phase; LA_bac_, left atrium before atrial contraction; LA_min_, left atrium minimum volume. **Reservoir function:** Total emptying volume (TEV) = LAmax-LAmin; Total emptying fraction = TEV/LAmax × 100. **Conduit function:** Passive emptying volume (PEV) = LAmax-LAmdv; Passive emptying fraction (PEF) = PEV/LAmax × 100; Conduit volume: Stroke volume-TEV. **Pump function:** Active emptying volume (AEV) = LAbac-LAmin; Active emptying fraction = AEV/LAmax × 100.

CMR is the reference standard for the measurement of the LV and LA volumes. Time/volume curves are generated from determining the total volume throughout the entire cardiac cycle on a complete short axis stack of the LV and LA. From the derivative, filling rates and fractions can be determined (Figure 4 and Figure 5). Manual data processing is time-consuming, but with automated software, this can be done immediately after the scan.

### 3.8. LV Time/Volume Curves—Early and Active Peak Filling Rate

Any part of a filling curve can be assessed, but most commonly, the early peak filling rate (ePFR) and occasionally the late or active/atrial peak filling rate (aPFR) are determined. With normal LV unwinding and compliance, the ePFR is high, and it decreases with diminishing LV unwinding and compliance (Figure 4). Several papers have reported normal values [36,37,38]. Maceira et al. [36] published the largest normal dataset (120 subjects of different ages; 50% women), and their study demonstrated a significant age-related decrease in ePFR (Table 5). The ePFR correlates with LVEDV (decreasing slightly with age); hence, the ePFR is often indexed to LVEDV, after which there is still a substantial difference between the young and elderly (Table 6) [36]. The ratio of ePFR to aPFR also decreases with age (Table 7) [36,39], but hypertension does not seem to have an additional effect on ePFR/aPFR in the elderly (Table 7) [39]. The response of ePFR to pharmacologic stress has been reported with dobutamine and glycopyrrolate by Ahtarovski et al. [40] in healthy young and elderly patients. During dobutamine stress, the peak-filling rate increased in both groups, but less so in the elderly (Table 5). With a glycopyrrolate-related increase in heart rate, ePFR increased in the young but decreased in the elderly (Table 5), suggesting that the response to glycopyrrolate stress may be an indicator of LV compliance. ePFR was decreased in patients with DM2 and IHD, but not in patients with hypertension or HCM [41,42,43] (Table 5 and Table 6).

ePFR/LVEDV is lowered in patients with IHD but not in patients with DM2 or HCM [41,42,43] (Table 5 and Table 6). In patients with hypertension, ePFR/LVEDV was only lowered in studies that had control groups with considerably lower age, which therefore must be considered inconclusive [32,41,44] (Table 5 and Table 6). In two studies, HFpEF patients when compared to controls have an increased pulmonary capillary wedge pressure, an increased E/e′ on echo, and a significantly lowered ePFR/LVEDV [15,16] (Table 6, Figure 2). A total of 101 patients with various diseases (all LVEF>55%) were assessed by echo-Doppler and CMR, and ePFR/aPFR was found to be lower in the patients with diastolic dysfunction as per 2009 ASE/EACI guidelines (Table 7) [45]. In contrast, in another study of 102 patients with a variety of diseases but normal systolic function and with reduced diastolic function according to 2009 ASE/EACI guidelines, no significant influence of echo-parameters was noted on ePFR (Table 5), and, of interest, ePFR/LVEDV was actually increased in patients with diastolic dysfunction (Table 6) [17]. The difference may be a matter of the analyzing technique, as the control group (patients with normal diastolic function) had an ePFR value that was considerably lowered compared to values from other studies. Furthermore, the group of control patients was slightly older than the studied subjects. Another study comparing CMR values to echo-determined diastolic dysfunction (2016 ASE/EAI guidelines) showed a reduced ePFR between grade I and II and controls, but no difference between grade III and controls, suggesting a pattern of pseudonormalization (Table 5, Figure 2) [14]. The ePFE/LVEDV was not presented. One small study reported an ePFR indexed to LV stroke volume divided by BSA in 13 patients with HCM. Interestingly, they found ePFR/LVSVi to be lower in patients with peak oxygen uptake below the median (median ePFR/LVSVi 5.12 m^2^/s IQR 4.16–6.82 vs. 7.98 IQR 7.46–8.21) [46].

### 3.9. LV Time/Volume Curve—Summarized

For early peak-filling rates, normal age-related values have been reported and must be taken into consideration in patient studies. Studies performed in patient groups with diseases often related to impaired filling have not been conclusive, and still, few studies exist with proper correlation to established echo or invasive parameters. In the well-controlled studies by Hieda et al. and Gao et al., in patients with HFpEF, ePFR/LVEDV was lowered [15,16]. Using the lowest ePFR/LVEDV estimate from the two studies, an ePFR/LVEDV below 2.1 ± 0.8 provided a cut-off value for significant diastolic dysfunction. Another study suggested that some degree of pseudonormalization may be a seen if ePFR is not related to LVEDV. In recent years, highly improved automatic analyzing software has become available; consequently, these parameters could now easily be assessed in a clinical setting. However, follow-up studies on the clinical impact of impaired filling rates and on appropriate cut-off values are still warranted.

### 3.10. LA Time/Volume Curve—Reservoir, Conduit, and Pump Function

Increased LA volume is well-established as a measure of longstanding high filling pressure secondary to increased LV end-diastolic pressure. When indexed to body surface area, four of the five studies found no gender or age-related difference in LA maximum volume or LA total emptying volume (Table 8 and Table 9) [47,48,49,50,51]. With ageing, however, the LA passive emptying volume and conduit volume decrease while the active emptying volume increases (Table 10 and Table 11) [52,53]. Functional parameters all demonstrate the age-related diastolic stiffening of the heart (Table 9, Table 10 and Table 11) [40,52,53]. During pharmacological stress with both dobutamine and glycopyrrolate, emptying fractions in the elderly decrease while they remain unchanged in the young, whereas atrial volumes remain unchanged (Table 9, Table 10 and Table 11) [40]. Eighteen papers studying different disease groups were found. In hypertensive patients, maximal LA volume is higher than in healthy subjects (Table 8) [54]. In patients with HCM, LA volumes are also higher, with lowered total and passive emptying fractions (Table 8, Table 9, Table 10 and Table 11) [19,55,56,57]. The results on the active emptying fraction are conflicting, with some studies showing an increase and some a decrease. Two studies on patients with DM2 found no differences in LA maximal volume or LA total emptying fraction compared to normal age-matched controls, but LA passive emptying fraction is lowered (Table 9 and Table 10) [58,59]. However, none of the patients in the above-mentioned studies had documented impaired filling. In a follow-up study of 536 patients with DM2, increased LA maximum volume, and decreasing LA total, passive and active emptying fractions were associated with a high incidence of cardiovascular disease even after adjusting for other risk factors [60]. In patients with cardiac amyloidosis, the maximal LA volume is high, but the LA total and active emptying fractions are lowered, probably reflecting amyloidosis of the LA wall with reduced LA pump function (Table 9, Table 10 and Table 11) [61,62]. Furthermore, in amyloidosis, echo-Doppler average e′ demonstrated a moderate correlation to LA total emptying fraction and active emptying fraction (estimates for echocardiography were, however, not presented) (Table 8, Table 9, Table 10 and Table 11) [61]. In a study on patients with suspected myocardial ischemia undergoing dobutamine stress, patients with the smallest change in LA passive emptying fraction (<10.8%) experienced a higher incidence of MACE (Table 10) [63]. Furthermore, in that study, the interobserver variability was assessed and found good for both volumes and functional parameters. The lowered LA total emptying fraction, but not LA maximum volume, was an independent marker of mortality in an 8-year follow-up of a normal population (HR 1.56, CI95% 1.32–1.87) [64]. In contrast, another study found that increased LA total volume was an independent predictor of death and, furthermore, that the hazard ratio for death increased with increasing LA dilation [65]. In a population of HFpEF patients, maximal LA volume and total LA emptying fraction were associated with increasing NT pro-BNP [66]. Two studies of patients with HCM and HFpEF (2007 ASE/EACVI criteria [67]) compared to healthy controls found that both groups had reduced LA total and passive emptying fraction compared to controls (Table 9, Table 10 and Table 11, Figure 2) [18,19]. In yet another study of patients with unexplained dyspnea, patients with HFpEF (LVEDP ≥ 16) had increased maximal LA volume [15] (Table 8). In contrast, in a study of patients with HFpEF compared to patients with various diseases but without heart failure, no differences were found in any LA volume parameter [68]. Furthermore, Aqauro et al. used the 2016 ASE/EACI echo-Doppler guidelines to classify the diastolic function in patients with various diseases [14] (Table 9). They found that the LA total emptying fraction was gradually lowered with increasing grades of diastolic dysfunction. The parameter could be used to successfully distinguish diastolic grade II and III from grade I and from patients with normal diastolic function. However, as presented in Table 9 and Figure 2, the estimates from Aqauro et al. diverged noticeably from other studies, being considerably lower. This is possibly explained by the fact that they performed the analysis on a short axis stack, whereas most other studies did the analysis using a biplane area-length method.

### 3.11. LA Time/Volume Curve—Summarized

CMR is a non-invasive reference standard for assessment of LA volume, and a number of relevant functional parameters can be assessed. Increased LA maximal volume (>52 mL/m^2^) and decreased LA total (<40%) and passive emptying fractions (<16%) seem good indicators of poor cardiovascular outcome. The parameters should be interpreted with caution in patients with disease of the atrium itself (notably amyloidosis). As a first approximation, based on two studies, a cut-off point for the dobutamine diastolic stress test could be a decrease in LA passive emptying fraction of >11. These parameters are also fast and easy to measure in a clinical setting.

### 3.12. Velocity-Encoded Phase-Contrast Sequences

LV mitral valve inflow can be assessed with CMR using phase-contrast velocity-mapping, in a manner similar to what is done with echo-Doppler. The parameters measured are usually early and late maximal inflow velocities, in combination with early and late maximal myocardial tissue relaxation velocity. Overall agreement with echo-Doppler has been assessed in several studies, and most studies find overall good agreement, but CMR parameters are generally lower in amplitude and underestimate several parameters [70,71,72,73,74,75,76,77,78,79], likely because of the lower temporal frame rate with CMR in comparison with echo-Doppler. Good agreement has been shown between CMR postero-septal E/é and pulmonary capillary wedge pressure in patients with hypertension-related LV hypertrophy [71], but other CMR E/é positions were not correlated to pulmonary capillary wedge pressure.

Asharafpoor et al. provided an overview of age-related normal reference values of blood and tissue velocity parameters [80]. In patients with DM2, early inflow, as well as the E/A-ratio, was lowered compared with age-matched controls [81,82,83,84]. In patients with cardiac amyloidosis, in general, echo-Doppler and CMR indices of blood and tissue velocity correlate well, but CMR again underestimated the early peak filling velocity, missing some of the patients with restrictive filling patterns on echo-Doppler [73]. In patients with AS-related LV hypertrophy, CMR velocity-encoded phase-contrast successfully identified patients with echo-Doppler diagnosed impaired diastolic function [77,85]. Furthermore, another study of AS patients showed that improvement of the diastolic function after valve replacement was detectable [86].

### 3.13. Velocity-Encoded Phase-Contrast Sequences—Summarized

In general, determining the E/A or E/e′ ratio with CMR is inferior to echo-Doppler as the lower temporal resolution of CMR may underestimate the early inflow velocity in particular. However, the method could be useful in patients where echo-Doppler is not feasible.

### 3.14. LV and LA Feature Tracking—Brief Description

From a standard short and longitudinal axis, SSFP cine images, LV, and LA strain are determined using post-processing software based on the identification and tracking of differences in the myocardial signal, or “speckles”, in the image. Global values for LV and LA can be used to assess relaxation and potentially compliance of the LV. The technique allows for analyses of longitudinal, circumferential, and radial diastolic strain rates of LV. For LA, the following parameters can be assessed: those related to LA reservoir function (“total diastolic strain and strain rate”; the sum of early and active diastolic strain); LA conduit function (early diastolic strain and peak early diastolic strain rate); and LA booster pump function (active diastolic strain and peak late diastolic strain rate).

### 3.15. LV Feature Tracking—Peak Diastolic Strain Rate

In total, five studies were identified. One larger study described normal values from 150 healthy subjects (21–71 years) [87]. All diastolic strain rates decrease with age, but normal age-related ranges overlap, and hence the pooled normal values seem to be the best first approximation for normal diastolic function (Table 12). In a study of patients with DM2, no differences were found in diastolic strain rates when compared to healthy controls (Table 12) [88]. One study compared CMR feature tracking with tagging in patients with AS and showed a significantly higher peak early diastolic circumferential strain with feature tracking (epicardial/endocardial average) of 1.29 ± 0.34 vs. 1.01 ± 0.31 with CMR tagging, and hence measures from the two methods were not directly comparable [31]. In 45 patients with HCM, patients had a peak early circumferential endocardial strain rate comparable with healthy subjects, but when normalized to the corresponding systolic strain, it was lowered, hence indicating that the diastolic relaxation phase was indeed comparatively prolonged (Table 12) [89]. In patients with HFpEF compared to controls, circumferential-myocardial strain rate was lowered (Table 12) [20].

### 3.16. LV Feature Tracking—Summarized

LV feature tracking is still a novel technique, but with promising potential and well-described normal values. The ability to assess strain on existing SSFP cine images and the easy analysis process are particularly appealing. However, few studies have been published so far, and thus conclusions should be drawn with caution. Furthermore, no studies have presented comparisons to echo-Doppler or invasive pressure measurements. The technique is fairly easy to post-process.

### 3.17. LA Feature Tracking—Reservoir, Conduit, and Pump Function

Comparable patterns are seen for reservoir and conduit strain with a decrease in most patient groups. For booster pump strain, generally, an increase was seen except for in patients with diseases also present in the atrium where a decrease often was seen. Consequently, only results from assessments of the reservoir function have been shown in tables. In healthy individuals, with age, reservoir, and conduit strain and corrosponding strain rate decreases, while there is no age-related difference in booster pump strain and strain rate (Table 13 and Table 14) [21,90]. In a study of 21 elderly healthy subjects, the scan-rescan reproducibility and inter-observer reducibility were good with coefficients of variance of 0.57% and 5.28%, respectively [91]. The sample size required for the detection of a 10–15% difference was then calculated as 7–15 patients. A comparable pattern to that found in the elderly was found in patients with HCM (Table 13 and Table 14) [55,57]. Furthermore, in HCM patients, a reduced total longitudinal strain <18% was associated with all-cause mortality and heart failure [57]. In this study, the intra- and inter-observer agreement was also good with coefficients of variance of 3% and 5.6%, respectively. A cutoff of 18.8% for total longitudinal strain was also identified to predict MACE in a cohort of patients with acute myocardial infarction [92]. In patients with hypertension, conduit strain and strain rate are impaired [93]. In patients with moderate-to-severe AS, total, early, and active strain and strain rates were all lowered when compared to healthy subjects (Table 13 and Table 14) [94]. In patients with DM2, 80% did not have impaired diastolic function on echo-Doppler, and furthermore, only conduit strain was reduced when compared to controls; all other strains and strain rates were similar to controls [95]. Two studies compared healthy controls to patients with HCM and to patients with HFpEF. All patients had higher LA maximal volume and reduced reservoir, conduit, and booster pump strain and strain rates compared to controls; however, patients were generally older than controls (Table 13 and Table 14) [18,19]. In another study of patients with HFpEF compared to controls (younger and with lower BMI and less hypertension), HFpEF patients had increased invasive LVEDP and impaired reservoir and conduit strain and strain rates but similar booster pump function (Table 13 and Table 14) [22].

### 3.18. LA Feature Tracking—Summarized

LA feature tracking is a promising technique, although, thus far, studies are few. Data processing is simple and fast, and reproducibility is good, making it an appealing technique. Reduced LA reservoir function seems to differentiate patients from healthy controls (Table 13, Figure 2). Estimates showed an age dependence which should be considered. The post-processing of the images is still under development and consequently not broadly available in a clinical setting.

### 3.19. Other Novel Techniques

Eighteen studies were identified with more novel and less well-established techniques, including atrioventricular motion assessment, LA transit time, blood velocity mapping, 4D flow kinetic energy, and vortex formation analysis [96,97,98,99,100,101,102,103,104,105,106,107,108,109,110,111,112,113]. With little comparison to other techniques and few data on how to use them to establish clinically relevant diastolic dysfunction, these techniques have not been reviewed in more detail.

## 4. Conclusions

Our systematic literature search revealed several promising CMR techniques for assessing LV diastolic dysfunction, but still very few studies were found with comparisons to established measures of diastolic dysfunction, notably invasive or echo-Doppler. Furthermore, few CMR studies exist with clinical outcomes. Tagging and feature tracking (both LV and LA strain measurements) hold promise, the former especially addressing the first ATP-dependent active LV relaxation, but studies are still few and require further investigation to improve the standardization of analysis and reporting and to establish age-related changes. For -dφ′/dV′ ≥6.2, both specificity and sensitivity are high for severely compromised LV diastolic filling. Initial promising findings will require further studies to improve standardization of analysis and reporting and to establish age-related changes. Due to the lower temporal resolution of CMR in comparison with echo-Doppler, CMR velocity-encoded phase contrast assessment of E/A or E/e′ ratios does not appear to offer any improvement over echo-Doppler unless an adequate echo-window cannot be obtained or unless internal validation of signs of diastolic dysfunction is needed in CMR studies performed for other reasons. For CMR to become of value in the study of diastolic function, it would be important to use it for the parameters for which it has a comparative advantage for improving echo-Doppler. For both LV filling and LA emptying parameters, age-related normal values are well-established and must be considered with respect to what normal aging-related stiffening of the LV can account for. An LV early peak filling rate (ePFR), especially when indexed to LVEDV, reflects diastolic dysfunction with abnormal function indicated by a value of <2.1 ± 0.8 s^−1^. Further changes of ePFR during diastolic stress testing with glycopyrrolate, demonstrated ePFR decreasing in the elderly but increasing in the young, underlining the usefulness of “diastolic stress-testing”. However, further studies including cardiovascular outcome and validation against established measurements of diastolic dysfunction are needed. For LA parameters, a maximal LA volume >52 mL/m^2^ and a lowered LA total (<40%) and passive emptying fractions (<16%) can be considered signs of diastolic dysfunction.

Conclusively, CMR, especially if incorporating the determination of LV myocardial mass and fibrosis, may offer the possibility of reclaiming the term “diastolic dysfunction” from “heart failure with preserved ejection fraction” in order to study the specific mechanisms and to explore mechanisms and treatments. However, in order to do so, further validation against well-established echo-Doppler and/or invasive determination of LV diastolic pressures or large clinical outcome studies are now firmly needed.

## Figures and Tables

**Table 1:** CMR tagging—diastolic rotation and untwisting rates.

Parameter	Author, Year	Controls, *n* (Age ^#^)	Patients, *n* (Age ^#^)	Sequence Parameter	Estimate, Controls	Estimate, Patients	Comparator ^^^
Normalized apical rotation rate ^◊^ (s^−1^)	Oxenham [24] 2003	15 (22 ± 3) Healthy young	15 (69 ± 4) Healthy elderly	1.5T SPAMM TR: 35–45 ms ST: 7 mm SR: In-plane 1 mm/pixel	−7 ± 1	−5 ± 1 *	Echo-Doppler; E 74 ± 16 vs. E 46 ± 10
-	Thompson [25] 2010	32 (33 ± 7) Healthy		1.5T SPAMM TR: 20 ms ST: 8 mm SR: Matrix 192 × 128 FoV:300–380 mm	−13 ± 3		No
-	Nagel [26] 2000	12 (29 ± 6) Healthy	13 (61 ± 12) AS	1.5T CSPAMM TR 35 ms ST: 6–8 mm SR: In-plane pixel 1.4 × 1.4 mm	−11 ± 2	−7 ± 1 *	No
Normalized global untwisting rate ^◊^ (s^−1^)	Thompson [25] 2010	32 (33 ± 7) Healthy		See above	−14 ± 2		No
-	Reyhan [27] 2013	13 (33 ± 11) Healthy		1.5T CSPAMM TR: NR ST: 5–6 mm SR: Matrix 192 × 144 FoV 300–360 × 280–300 mm	−8 ± 2		No
-	Reyhan [28] 2014	13 (33 ± 11) Healthy		1.5T CSPAMM TR: NR ST: 5–6 mm SR: Matrix 192 × 144 FoV 300–360 × 280–300	−9 ± 2		No

^#^ Age was presented as mean and standard deviation. ^^^ Presented as controls vs. patients. ^◊^ Normalized to peak systolic absolute value, e.g., rotation or twist as appropriate. * *p* < 0.05 vs. control. AS, Aortic stenosis; E, early mitral diastolic inflow velocity; T, Tesla; SPAMM, spatial modulation of magnetization; CSPAMM, complementary spatial modulation of magnetization; TR, temporal resolution; ST, slice thickness; SR, spatial resolution; FoV, field of view; NR, not reported.

**Table 2:** CMR tagging—diastolic strain rates.

Parameter	Author, Year	Controls, *n* (Age ^#^)	Patients, *n* (Age ^#^)	Sequence Parameter	Estimate, Controls	Estimate, Patients	Comparator ^^^
Global circumferential-longitudinal shear strain rate (% s^−1^)	Thompson [25] 2010	32 (33 ± 7) Healthy		1.5T SPAMM TR: 20 ms ST: 8 mm SR: Matrix 192 × 128 FoV:300–380 mm	72 ± 13		No
Normalized global circumferential-longitudinal shear strain rate ^◊^ (s^−1^)	Thompson [25] 2010	32 (33 ± 7) Healthy		See above	−12 ± 1.4		No
Global peak circumferential strain rate (% s^−1^)	Fonseca [29] 2004	31 (47 ± 24) Healthy	28 (53 ± 8) DM2	1.5T SPAMM TR: 35–45 ms ST:8 mm SR: In-plane 1 mm/pixel	108 ± 41	71 ± 20 *	E/A 1.3 ± 0.6 vs. 0.9 ± 0.2
-	Singh [30] 2016		15 (66 ± 10) Moderate to severe AS	3T SPAMM TR: 46 ST: 8 mm SR: NR		79 ± 15	Lateral E/e′ 11 ± 3
-	Singh [31] 2015		8 (67 ± 8) 10 (67 ± 9) Moderate-Severe AS	1.5T & 3T CSAPMM & SPAMM TR: 42 & 46 ST: 6&8 mm SR: NR		100 ± 31 82 ± 26	No
-	Schiros [32] 2014	40 (42 ± 13) Healthy	60 (55 ± 12) AH	1.5T SPAMM TR: NR ST: 8 mm SR: matrix 256 × 128 FoV 40 × 40	101 ± 28	79 ± 27 *	No
Peak mid-ventricular strain rate (% s^−1^)	Musa [33] 2017	52 (81 ± 6) Severe AS		1.5T CSPAMM TR: NR ST: 10 mm SR: matrix 128 × 128 FoV 300 mm		2.2 ± 1.5	LVEDP; see text
Peak Longitudinal strain rate (% s^−1^)	Fonseca [29] 2004	31 (47 ± 24) Healthy	28 (53 ± 8) AM2	See above	92 ± 37	63 ± 2	See above
-	Schiros [32] 2014	40 (42 ± 13) Healthy	60 (55 ± 12) AH	See above	104 ± 32	79 ± 30	No

^#^ Age was presented as mean ± SD. ^^^ Presented as controls vs. patients. ^◊^ Normalized to peak systolic absolute value, e.g., rotation or twist as appropriate. * *p* < 0.05 vs. control. DM2, diabetes mellitus type 2; AH, arterial hypertension; AS, aortic stenosis; E, early mitral diastolic inflow velocity; A, active mitral diastolic inflow velocity; e′, early diastolic mitral annular velocity; T, Tesla; SPAMM, spatial modulation of magnetization; CSPAMM, complementary spatial modulation of magnetization; TR, temporal resolution; ST, slice thickness; SR, spatial resolution; NR, not reported.

**Table 3:** CMR tagging—time to peak untwisting rate.

Parameter	Author, Year	Control, *n* (Age ^#^)	Patients, *n* (Age ^#^)	Sequence Parameter	Estimate, Controls	Estimate, Patients	Comparator
Time to peak untwist	Stuber [12] 1999	11 (34 ± 9) Healthy	12 (58 ± 13) AS	1.5T CSPAMM TR:35 ms ST: 6 mm SR: matrix 256 × 256 FoV 360 mm	47 ± 23 ms	88 ± 19 ms *	Invasive (Figure 2)
-	Nagel [26] 2000	12 (29 ± 6) Healthy	13 (61 ± 12) AS	1.5T CSPAMM TR 35 ms ST: 6–8 mm SR: In-plane pixel 1.4 × 1.4 mm	56 ± 25 ms	103 ± 28 ms *	No

^#^ Age was presented as mean and standard deviation. * *p* < 0.05 vs. control. AS, Aortic stenosis; LVEDP, left ventricle end-diastolic pressure, T, Telsa; CSPAMM, complementary spatial modulation of magnetization; TR, temporal resolution; ST, slice thickness; SR, spatial resolution.

**Table 4:** CMR tagging—peak change of the torsion shear angle versus volume curve in the early diastole (−dφ′/dV′).

Parameter	Author, Year	Control, *n* (Age ^#^)	Patients, *n* (Age ^#^)	Sequence Parameter	Estimate, Controls	Estimate, Patients	Comparator ^^^
Normalized peak early torsion shear angle vs. volume change ^◊^	Sharifov [13] 2015	18 (62 ± 5) Patients^∆^ with low invasive LV pressure	18 (60 ± 8) Patients ^∆^ with high invasive LV pressure	1.5T SPAMM TR: NR ST: 8 mm SR: matrix 256 × 128 FoV 40	3.6 ± 1.6	7.6 ± 4.2 *	Invasive (Figure 2)
^-^	Schiros [32] 2014	40 (42 ± 13) Healthy	60 (55 ± 12) AH	1.5T SPAMM TR: NR ST: 8 mm SR: matrix 256 × 128 FoV 40 × 40	6.3 ± 3.9	10.5 ± 8.5 *	No

^#^ Age was presented as mean ± SD. ^ Presented as controls vs. patients. ^◊^ Normalized to maximum values, e.g., angle or volume as appropriate. * *p* < 0.05 vs. control. ^∆^ Patients with no acute myocardial infarction undergoing coronary angiography for chest pain or dyspnea. AH, arterial hypertension; Tau, the time constant of LV diastolic relaxation; T, Tesla; SPAMM, spatial modulation of magnetization; TR, temporal resolution; ST, slice thickness; SR, spatial resolution; NR, not reported.

**Table 5:** LV time/volume curves—early peak filling rate.

Parameter	Author, Year	Controls, *n* (Age ^#^)	Patients, *n* (Age ^#^)	Sequence Parameters	Estimate, Controls	Estimate, Patients	Comparator ^^^
ePFR (mL s^−1^)	Maceira [36] 2006	20 (20–29), Healthy young	20 (70–79), Healthy elderly	1.5T TR: 22 ± 1 ms ST:7 mm SR: In-plane pixel size 2.1 × 1.3 mm Phases: NR	720 ± 143	276 ± 143 *	No
-	Aquaro [14] 2018	20 (51 ± 16), Healthy. Normal DD ^◊^.	40 (54 ± 18), Reduced DD ^◊^. Various diseases	1.5T TR: NR ST: 8 mm SR: matrix 224 × 224 FoV 400 mm Phases: 30	375 ± 63	DD I: 247 ± 47 * DD II: 325 ± 92 * DD III: 353 ± 92	Echo-Doppler (Figure 2)
-	Gao [15] 2019	26 (65 ± 10), Patients with unexplained dyspnea	25 (69 ± 8), HFpEF	3T TR: NR ST:8 mm SR: matrix 232 × 219 FoV: NR Phases: NR	253 ± 63	222 ± 66	LVEDP < 16, E/e′ 9.4 ± 1.4 vs. LVEDP ≥ 16, E/e′ 12.7 ± 3.1
-	Graca [30] 2014	21 (55 ± 7), Normo-glycemic controls	41 (58 ± 7), DM2	3T TR:25–40 ms ST:8 mm SR: Matrix 256 × 156 Pixel size 2.1 × 1.6 mm Phases: 25	376 ± 103	293 ± 52 *	No
-	Chacho [31] 2016	20 (44–56), Healthy	41 (45–57), HCM 21 (47–61), AH	1.5T TR: 50 ms ST:8 mm SR: Matrix 256 × 256 FoV 280–340 mm Phases: 25	445 (372–532)	HCM 414 (349–536) AH 395 (356–528)	No
-	Rodriguez-Granillo [29] 2012	25 (57 ± 15) Healthy	25 (62 ± 12) IHD	3T TR:49.3 ms ST: 8 mm SR: matrix 144 × 157 FoV 320 mm Phases 30	316 ± 126	252 ± 98 *	No
-	Nacif [37] 2016	66 (67 ± 9) Normal DD ^◊◊^	15 (64 ± 10) Reduced DD ^◊◊^	1.5T TR: 30 ± 5 ms ST:8 mm SR: Matrix 205 × 256 FoV 360 mm Phases 30	189 ± 66	214 ± 72	Average E/e′ 7.3 ± 1.8 vs. 14.2 ± 5.4
Dobutamine stress ePFR	Ahtarovski [26] 2012	20 (20–30) Healthy young	20 (60–70) Healthy Elderly	1.5T TR:NR ST:7 mm SR: Matrix 192 × 162 FoV 300–360 mm Phases 25	Increase by 72 ± 24%	Increase by 20 ± 9% *	No
Glycol-pyrrolate stress ePFR	Ahtarovski [26] 2012	20 (20–30) Healthy young	20 (60–70) Healthy elderly	See above	Increase by 22 ± 10%	Decrease by −13 ± 9% *	No

^#^ Age was presented as mean ± SD. ^^^ Presented as controls vs. patients. * *p* < 0.05 vs. control. ^◊^ Patients with various diseases but with normal vs. reduced diastolic function according to 2016 ASE/EACI guidelines. ^◊◊^ Patients with various diseases but with normal vs. reduced diastolic function according to 2009 ASE/EACI guidelines. ePFR, early peak filling rate; DM2, diabetes mellitus type 2; HCM, hypertrophic cardiomyopathy; AH, arterial hypertension; IHD, ischemic heart disease; DD, diastolic dysfunction; E, early mitral diastolic inflow velocity; e′, early diastolic mitral annular velocity; T, Tesla; TR, temporal resolution; ST, slice thickness; SR, spatial resolution; NR: Not reported.

**Table 6:** LV time/volume curves—early peak filling rate indexed to LV end-diastolic volume.

Parameter	Author, Year	Controls, *n* (Age ^#^)	Patients, *n* (Age ^#^)	Sequence Parameter	Estimate, Controls	Estimate, Patients	Comparator
ePFR/ LVEDV (s^−1^)	Maceira [36] 2006	20 (20–29) Healthy young	20 (70–79) Healthy elderly	1.5T TR: 22 ± 1 ms ST:7 mm SR: In-plane pixel size 2.1 × 1.3 mm Phases: NR	4.8 ± 0.8	2.3 ± 0.8 *	No
-	Schiros [32] 2014	40 (42 ± 13) Healthy	60 (55 ± 12) AH	1.5T TR: NR ST:8 mm SR: matrix 256 × 128 FoV 40 × 40 cm Phases: 20	3.0 ± 0.6	2.5 ± 0.7 *	No
-	Gupta [44] 2015	45 (41 ± 13) Healthy	15 (54 ± 6) AH	1.5T TR: NR ST:8 mm SR: matrix 256 × 128 FoV 40 × 40 cm Phases 20	3.1 ± 0.6	2.2 ± 0.8 *	No
-	Rodriguez-Granillo [43] 2012	25 (57 ± 15) Healthy	25 (62 ± 12) IHD	3T TR:49.3 ms ST: 8 mm SR: matrix 144 × 157 FoV 320 mm Phases 30	3.3 ± 1.5	1.6 ± 1.2 *	No
-	Gao [15] 2019	26 (65 ± 10), Patients with unexplained dyspnea	25 (69 ± 8), HFpEF	3.0T TR: NR ST:8 mm SR: matrix 232 × 219 FoV: NR Phases: NR	2.6 ± 0.8	2.1 ± 0.8 *	Echo-Doppler. (Figure 2)
-	Hieda [16] 2017	12 (65–77) Healthy	10 (62–85) HFpEF	1.5T TR:39 ms ST: NR SR: NR Phases: NR	3.6 ± 0.9	2.7 ± 0.9 *	Invasive and echo-Doppler (Figure 2)
-	Chacho [41] 2016	20 (44–56) Healthy	HCM 41 (45–57) AH 21 (47–61)	1.5T TR: 50 ms ST:8 mm SR: Matrix 256 × 256 FoV 28–34 × 28–34 cm Phases: 25	2.9 (2.6–3.2)	HCM 2.7 (2.3–3.3), AH 2.7 (2.2–3.1)	No
-	Graca [42] 2014	21 (55 ± 7) Healthy	41 (58 ± 7) DM2	3T TR 25–40 ms ST:8 mm SR: matrix 256 × 156 Pixel size 2.1 × 1.6 mm Phases 25	3.2 ± 0.8	3.1 ± 0.7	No
-	Nacif [17] 2016	66 (67 ± 9) Normal DD ^◊◊^	15 (64 ± 10) Reduced DD ^◊^	1.5T TR: 30 ± 5 ms ST:8 mm SR: Matrix 205 × 256 FoV 36 cm Phases: 30	1.8 ± 0.5	2.1 ± 0.4 *	Echo-Doppler: (Figure 2)

^#^ Age was presented as mean ± SD. * *p* < 0.05 vs. control. ^◊◊^ Patients with various diseases but with normal vs. reduced diastolic function according to 2009 ASE/EACI guidelines. ePFR, early peak filling rate; LVEDV, left ventricular end-diastolic volume; AH, arterial hypertension; IHD, ischemic heart disease; HFpEF, heart failure with preserved ejection fraction; HCM, hypertrophic cardiomyopathy; DM2, diabetes mellitus type 2; DD, diastolic dysfunction; T, Tesla; TR, temporal resolution; ST, slice thickness; SR, spatial resolution; NR, not reported.

**Table 7:** LV time/volume curves—early peak filling rate indexed to active peak filling rate.

Parameter	Author, Year	*n* (Age ^#^), Controls	*n* (Age ^#^), Patients	Sequence Parameter	Estimate, Controls	Estimate, Patients	Comparator ^^^
ePFR/ aPFR	Maceira [36] 2006	20 (20–29) Healthy young	20 (70–79) Healthy elderly	1.5T TR: 22 ± 1 ms ST:7 mm SR: In-plane pixel 2.1 × 1.3 mm Phases: NR	3.0 ± 0.34	0.5 ± 0.34*	No
	Parikh [39] 2017	16 (60–69) Healthy	15 (60–69) AH	3T TR: NR ST: NR SR: NR Phases: NR	1.5 ± 0.7	1.4 ± 0.4	No
	Kawaji [45] 2009	51 (41 ± 14) Normal DD ^◊◊^	50 (64 ± 14) Reduced DD ^◊^	1.5T TR: 36 ± 10 ST: 6 mm SR: in-plane pixel 1.9 × 1.4 mm Phases: NR	3.1 ± 1.6	1.6 ± 1.1 *	Average E/e′ 7 ± 3 vs. 15 ± 10

^#^ Age was presented as mean ± SD. ^^^ Presented as controls vs. patients. * *p* < 0.05 vs. control. ^◊◊^ Patients with various diseases but with normal vs. reduced diastolic function according to 2009 ASE/EACI guidelines. ePFR, early peak filling rate; aPFR, active peak filling rate; AH, arterial hypertension; E, early mitral diastolic inflow velocity; e′, early diastolic mitral annular velocity; T, Tesla; TR, temporal resolution; ST, slice thickness, SR; spatial resolution; NT, Not reported.

**Table 8:** LA time/volume curves—maximum LA volume.

Parameter	Author, Year	*n* (Age ^#^), Controls	*n* (Age ^#^), Patients	Sequence Parameter	Estimate, Controls	Estimate, Patients	Comparator ^^^
LA maximum volume/ BSA (mL m^−2^)	Maceira [49] 2010	120 (20–80) Healthy		1.5T TR: 21 ± 1 ST:5 mm SR: in-plane pixel 2.1 × 1.3 mm Phases: NR From Short axis	40 ± 7		No
-	Khan [65] 2019	85 (39 ± 12) Healthy	10,890 (48–60) Various diseases	For controls 1.5T or 3T TR: NR ST: 6 mm SR: in-plan 1.5 × 1.5 × 2.1 mm Phases: 25–30 From 2 ch and 4 ch	21–52	LA dilatation Mild 52–62 Moderate 63–73 Severe >73	No
-	Gao [15] 2019	26 (65 ± 10), Patients with unexplained dyspnea	25 (69 ± 8), HFpEF	3T TR: NR ST:8 mm SR: matrix 232 × 219 FoV; NR Phases: NR From: NR	36 ± 12	46 ± 12 *	LVEDP < 16, E/e′ 9.4 ± 1.4 vs. LVEDP ≥ 16, E/e′ 12.7 ± 3.1
-	Janwanishst-aporn [54] 2016		111 (71 ± 10) AH	1.5T TR: NR ST: NR SR:1.25 × 1.25 × 8 mm^2^ Phases 25 From 2 ch and 4 ch		55 ± 16	No
-	Kowallick [55] 2016	23 (55 ± 11) Healthy	73 (59 ± 13) HCM	1.5T or 3T TR: 25–35 ms ST: NR SR: NR Phases 30 From 2 ch and 4 ch	38 ± 7	52 ± 12 *	No
-	Shang [59] 2017	35 (52 ± 13) Healthy	50 (55 ± 9) DM2	3T TR: NR ST:NR SR: NR Phases: NR From 2 ch and 4 ch	35 ± 12	38 ± 11	No
-	Kwong [61] 2015	37 (59 IQR 21) AH	22 (66 IQR 17) Amyloidosis	1.5T TR: 46 ST: 8 mm SR: in-plane pixel 1.5 × 1.8 or 1.8 × 2.1 mm Phases: NR From 2 ch and 4 ch	46 IQR 47	60 IQR 17 *	Echo-Doppler: See text
-	Hinojar [57] 2019	75 (53 ± 16) Various diseases but none cardiac	75 (55 ± 15) HCM	1.5T TR: NR ST: NR SR: 1.8 × 1.8 × 8 mm Phases: NR From: NR	44 ± 10	63 ± 20	No

^#^ Age shown as mean ± SD or range or median and IQR. ^^^ Presented as controls vs. patients. * *p* < 0.05 vs. control. LA, left atrium; BSA, body surface area; AH, arterial hypertension; HCM, hypertrophic cardiomyopathy; DM2, diabetes mellitus type 2; IQR, interquartile range; E, early mitral diastolic inflow velocity; e′, early diastolic mitral annular velocity; T, Tesla; TR, temporal resolution; ST, slice thickness; SR, spatial resolution; NR; not reported.

**Table 9:** LA time/volume curves—reservoir function.

Parameter	Author, Year	*n* (Age ^#^), Controls	*n* (Age ^#^), Patients	Sequence Parameter	Estimate, Controls	Estimate, Patients	Comparator
LA total emptying fraction (%)	Maceira [53] 2016	20 (20–29) Healthy young	20 (70–79) Healthy elderly	1.5T TR: 21 ± 1 ST:5 mm SR: IN-plane pixel 2.1 × 1.3 mm Phases: NR From Short axis	62 ± 6	55 ± 5 *	No
-	Aquaro [14] 2018	20 (51 ± 16), Healthy. Normal DD ^◊^.	40 (54 ± 18), reduced DD ^◊^. Various diseases	1.5T TR: NR ST: 8 mm SR: matrix 224 × 224 FoV 400 mm Phases: 30 From short axis	48 ± 5	DD I: 44 ± 5 * DD II: 25 ± 8 * DD III: 18 ± 12 *	Echo-Doppler (Figure 2)
-	Leng [19] 2018	50 (56 ± 13) Healthy	30 (55 ± 14) HCM 30 (62 ± 11) HFpEF^◊◊^	3.0T TR: NR ST: 8 mm SR: matrix 240 × 240 FoV 300 mm Phases: 30–40 From 2 ch and 4 ch	59 ± 5	HCM 51 ± 7 * HFpEF 48 ± 7 *	Figure 2
-	Kowallick [55] 2016	23 (55 ± 11) Healthy	73 (59 ± 13) HCM	See Table 8	59 ± 6	51 ± 12 *	No
-	Shang [59] 2017	35 (52 ± 13) Healthy	50 (55 ± 9) DM2	See Table 8	59 ± 8	52 ± 9	No
-	Kwong [61] (2015)	37 (59 IQR 21) AH	22 (66 IQR 17) Amyloidosis	See Table 8	40 ± 14	19 ± 14 *	Echo-Doppler: See text
-	Kowallick [18] 2014	10 (23–51) Healthy	10 (44–73) HCM 10 (58–82) HfpEF ^◊^	1.5T TR: NR ST: 6–8 mm SR: Matrix 192–256 × 164–220 FoV 260–400 × 230 × 340 Phases: NR From 2 ch × 4 ch	61 ± 6	59 ± 6 53 ± 7 *	Figure 2
-	Hinojar [57] 2019	75 (53 ± 16) Various diseases but none cardiac	75 (55 ± 15) HCM	See Table 8	55 ± 9	40 ± 16 *	No

^#^ Age shown as mean ± SD or range or median and IQR. * *p* < 0.05 vs. control. ^◊◊^ Signs of HF based on the modified Framingham criteria. ^◊^ HFpEF had diastolic dysfunction after 2007 criteria [67]. LA, left atrium; BSA, body surface area; HCM, hypertrophic cardiomyopathy; DM2, diabetes mellitus type 2; IQR, interquartile range; AH, arterial hypertension; HFpEF; heart failure with preserved ejection fraction; E, early mitral diastolic inflow velocity; e′, early diastolic mitral annular velocity; T, Tesla; TR, temporal resolution; ST, slice thickness; SR, spatial resolution; NT, not reported.

**Table 10:** LA time/volume curves—conduit function.

Parameter	Author, Year	Controls, *n* (Age ^#^)	Patients, *n* (Age ^#^)	Sequence Parameter	Estimate, Controls	Estimate, Patients	Comparator
LA passive emptying fraction (%)	Maceira [53] 2016	20 (20–29) Healthy young	20 (70–79) Healthy elderly	See Table 9	47 ± 6	29 ± 6 *	No
-	Kowallick [55] 2016	23 (55 ± 11) Healthy	73 (59 ± 13) HCM	See Table 8	32 ± 7	22 ± 10 *	No
-	Shang [59] 2017	35 (52 ± 13) Healthy	50 (55 ± 9) DM2	See Table 8	39 ± 11	29 ± 10 *	No
-	Kwong [61] (2015)	37 (59 IQR 21) AH	22 (66 IQR 17) Amyloidosis	See Table 8	18 ± 12	11 ± 13	Echo-Doppler: See text
-	Kowallick [18] 2014	10 (23–51) Healthy	10 (44–73) HCM 10 (58–82) HFpEF^◊^	See Table 9	35 ± 9	HCM 26 ± 6 * HFpEF 24 ± 4 *	Figure 2
-	Leng [19] 2018	50 (56 ± 13) Healthy	30 (55 ± 14) HCM 30 (62 ± 11) HFpEF^◊◊^	See Table 9	27 ± 7	HCM 24 ± 7 * HFpEF 19 ± 7 *	Figure 2
-	Hinojar [57] 2019	75 (53 ± 16) Various diseases but none cardiac	75 (55 ± 15) HCM	See Table 8	24 ± 13	16 ± 11	No
During dobutamine (%)	Ahtarovski [40] 2012	20 (20–30) Healthy Young	20 (60–70) Healthy elderly	1.5T TR:NR ST: 7 mm SR: Matix 192 × 162 FoV 300–360 mm Phases: 25 From Short axis	No change	22 ± 9 * Decreased from 30 ± 7	No
-	Farzaneh-Far [63] 2011		108 (61 ± 12) Low vs. high MACE incidence	1.5T TR:45–50 ms ST: 8 mm SR: In-plane pixel 1.5 × 2 mm Phases: NR From 2 ch and 4 ch		Decrease of >11 from rest *	No
During glycol-pyrrolate (%)	Ahtarov-ski [40] 2012	20 (20–30) Healthy young	20 (60–70) Healthy elderly	See above	No change	16 ± 9 * Decrease from 30 ± 7	No

^#^ Age shown as mean ± SD or range or median and IQR. * *p* < 0.05 vs. control. ^◊^ HFpEF had diastolic dysfunction after 2007 criteria [67]. ^◊◊^ Signs of HF based on the modified Framingham criteria [69]. LA, left atrium; BSA, body surface area; HCM, hypertrophic cardiomyopathy; DM2, diabetes mellitus type 2; IQR, interquartile range; AH, arterial hypertension; HFpEF, heart failure with preserved ejection fraction; MACE, major adverse cardiac events; T, Telsa; TR, temporal resolution; ST, slice thickness; SR, spatial resolution.

**Table 11:** LA time/volume curves—pump function.

Parameter	Author, Year	Controls, *n* (Age ^#^)	Patients, *n* (Age ^#^)	Sequence Parameter	Estimate, Controls	Estimate, Patients	Comparator
LA active emptying fraction (%)	Maceira [53] 2016	20 (20–29) Healthy young	20 (70–79) Healthy elderly	See above	32 ± 7	39 ± 7 *	No
-	Kowallick [55] 2016	23 (55 ± 11) Healthy	73 (59 ± 13) HCM	1.5T or 3T TR: 25–35 ms ST: NR SR: NR Phases: 30 From VLA and 4 ch	40 ± 8	38 ± 10	No
-	Kwong [61] (2015)	37 (59 IQR 21) AH	22 (66 IQR 17) Amyloidosis	1.5T TR: 46 ST: 8 mm SR: in-plane pixel 1.5 × 1.8 or 1.8 × 2.1 mm Phases: NR From 2 ch × 4 ch	28 ± 13	10 ± 11 *	Echo-Doppler: See text
-	Kowallick [18] 2014	10 (23–51) Healthy	10 (44–73) HCM 10 (58–82) HFpEF	1.5T TR: NR ST: 6–8 mm SR: Matrix 192–256 × 164–220 FoV 260–400 × 230 × 340 Phases: NR From 2 ch × 4 ch	26 ± 7	HCM 34 ± 6 * HFpEF 29 ± 7	No
-	Leng [19] 2018	50 (56 ± 13)	30 (55 ± 14) HCM 30 (62 ± 11) HFpEF	3.0T TR: NR ST: 8 mm SR: matrix 240 × 240 FoV 300 mm Phases:30–40 From 2 ch and 4 ch	43 ± 8	HCM 36 ± 7 * HFpEF 36 ± 7 *	No
-	Hinojar [57] 2019	75 (53 ± 16) Various diseases but none cardiac	75 (55 ± 15) HCM	1.5T TR: NR ST: NR SR: 1.8 × 1.8 × 8 mm Phases: NR From: NR	41 ± 11	27 ± 14	No

^#^ Age shown as mean ± SD or range or median and IQR. * *p* < 0.05 vs. control. LA, left atrium; BSA, body surface area; HCM, hypertrophic cardiomyopathy; IQR, interquartile range; AH, arterial hypertension; HFpEF, heart failure with preserved ejection fraction; T, Telsa; TR, temporal resolution; ST, slice thickness; SR, spatial resolution; NR, not reported.

**Table 12:** LV feature tracking—peak diastolic strain rate.

Parameter	Author, Year	Controls, *n* (Age ^#^),	Patients, *n* (Age ^#^)	Sequence Parameter	Estimate, Controls	Estimate, Patients	Comparator
Radial (s^−1^)	Andre [87] 2015	150 (46 ± 14) Healthy		1.5T TR: NR ST: 8 mm SR: In-plane pixel 2.2 × 2.2 mm Phases 35 From Short axis stack	−2.1 ± 0.5		No
-	Shang [88] 2019	36 (51 ± 12) Healthy	53 (54 ± 8) DM2	3T TR: NR ST: 6 mm SR: matrix 179 × 256 FoV 325 × 400 mm^2^ Phases 25 From 4 ch	−2.8 ± 1.0	−2.7 ± 0.9	No
Circumferential-endocardial (s^−1^)	Andre [87] 2015	150 (46 ± 14) Healthy		See above	2.1 ± 0.6		No
-	Nucifora [89] 2015	15 (46 ± 12) Healthy	45 (48 ± 17) HCM	1.5T TR:30 ms ST: 8 mm SR: matrix 205 × 256 FoV 340 × 340 mm Phases: NR from: NR	1.5 ± 0.8	1.5 ± 0.3	No
Circumferential-myocardial ^◊^ (s^−1^)	Andre [87] 2015	150 (46 ± 14) Healthy		See above	1.7 ± 0.5		No
-	Singh [31] 2015		8 (67 ± 8) 10 (67 ± 9) AS	1.5T & 3T CSAPMM & SPAMM TR: 42 & 46 ST: 6 & 8 mm SR: NR Phases: NR from: Apical, mid-ventricular and basal slice		1.3 ± 0.3	Tagging CMR; 1.0 ± 0.3
-	Mahmod [20] (2018)	14 (69 ± 6) Healthy	27 (72 ± 7) HFpEF	3T TR: NR ST:NR SR:NR Phases: NR from: Short axis stack	110 ± 28 % s^−1^	85 ± 27 % s^−1^ *	Figure 2
-	Shang [88] 2019	36 (51 ± 12) Healthy	53 (54 ± 8) DM2	3T TR: NR ST: 6 mm SR: matrix 179 × 256 FoV 325 × 400 mm^2^ Phases 25 From mid-ventricular short axis slice	1.4 ± 0.4	1.3 ± 0.4	No
Longitudinal-endocardial (s^−1^)	Andre [87] 2015	150 (46 ± 14) Healthy		See above. But from: 4 ch	1.8 (1.5–2.2)		No
Longitudinal-myocardial ^◊^ (s^−1^)	Andre [87] 2015	150 (46 ± 14) Healthy		See above. But from: 4 ch	1.6 (1.4–2.0)		No
-	Shang [88] 2019	36 (51 ± 12) Healthy	53 (54 ± 8) DM2	See above. But from: 4 ch	1.2 ± 0.3	1.1 ± 0.2 *	No
Normalized Circumferential-endocardial (s^−1^)	Nucifora [89] 2015	15 (46 ± 12) Healthy	45 (48 ± 17) HCM	See Above	−1.1 ± 0.2	−0.9 ± 0.3 *	No

^#^ Age shown as mean ± SD * *p* < 0.05 vs. control. ^◊^ Myocardial meaning, average using both endocardial and epicardial borders. LV, left ventricle; HCM, hypertrophic cardiomyopathy; T, Telsa; TR, temporal resolution; ST, slice thickness; SR, spatial resolution; NR, not reported.

**Table 13:** LA feature tracking—total longitudinal strain.

Parameter	Author, Year	Controls, *n* (Age ^#^)	Patients, *n* (Age ^#^)	Sequence Parameter	Estimate, Controls	Estimate, Patients	Comparator
Total longitudinal strain (%)	Evin [21] 2016	28 (25 ± 3) Healthy young	30 (59 ± 7) Healthy elderly	1.5T TR: 20–30 ms ST: 8 mm SR: matrix 260 × 192 FoV: NR Phases: NR	26 ± 6	21 ± 6 *	Figure 2
-	Kowallick [18] 2014	10 (23–51) Healthy	10 (44–73) HCM 10 (58–82) HFpEF^◊^	1.5T TR: NR ST: 6–8 mm SR: Matrix 192–256 × 164–220 FoV 260–400 × 230 × 340 Phases: NR	29 ± 5	22 ± 6* HCM 16 ± 6 * HFpEF	Figure 2
-	Evin [94] 2015	10 (64 ± 6) Healthy	10 (73 ± 15) AS	See above	23 ± 5	12 ± 7 *	No
-	Leng [19] 2018	50 (56 ± 13) Healthy	30 (55 ± 14) HCM 30 (62 ± 11) HFpEF	3.0T TR: ST: 8 mm SR: matrix 240 × 240 FoV 300 mm Phases 30–40	35 ± 5	HCM 27 ± 5 * HFpEF 24 ± 5 *	No
-	Von Roeder [22] 2017	12 (58 ± 9) Various diseases	22 (65 ± 9) HFpEF^◊^	1.5T TR: NR ST: 8–10 mm SR: Voxel size 1.25 × 1.25 × 8 mm^2^ Phases: NR	29 ± 6	22 ± 7	Figure 2
-	Hinojar [57] 2019	75 (53 ± 16) Various diseases	75 (55 ± 15) HCM	1.5T TR: NR ST: NR SR: 1.8 × 1.8 × 8 mm Phases: NR	30 ± 6	17 ± 8	No

^#^ Age shown as mean ± SD or range * *p* < 0.05 vs. control. ^◊^ By the 2007 ASE/EACVI guidelines [67]. LA, left atrium; HCM, hypertrophic cardiomyopathy; HFpEF, heart failure with preserved ejection fraction; AS, aortic valve stenosis; T, Telsa; TR, temporal resolution; ST, slice thickness; SR, spatial resolution; NR, not reported.

**Table 14:** LA feature tracking—total longitudinal strain rate.

Parameter	Author, Year	Controls, *n* (Age ^#^)	Patients, *n* (Age ^#^)	Sequence Parameter	Estimate, Controls	Estimate, Patients	Comparator
Total longitudinal strain rate (% s^−1^)	Evin [21] 2016	28 (25 ± 3) Healthy young	30 (59 ± 7) Healthy elderly	See Table 13	1.3 ± 0.4	0.9 ± 0.2*	E/e′ 5 ± 1 vs. 7 ± 2
-	Kowallick [18] 2014	10 (23–51) Healhy	10 (44–73) HCM 10 (58–82) HFpEF	See Table 13	1.1 ± 0.2	0.9 ± 0.2 * 0.8 ± 0.3 *	No
-	Evin [94] 2015	10 (64 ± 6) Healthy elderly	10 (73 ± 15) AS	See Table 13	1.5 ± 0.6	0.7 ± 0.5 *	No
-	Leng [19] 2018	50 (56 ± 13) Healthy	30 (55 ± 14) HCM 30 (62 ± 11) HFpEF	See Table 13	1.8 ± 0.4	HCM 1.3 ± 0.3 * HFpEF 1.1 ± 0.2 *	No
-	Von Roeder [22] 2017	12 (58 ± 9) Various diseases	22 (65 ± 9) HFpEF ◊	See Table 13	1.1 ± 0.3	0.8 ± 0.3	E/e′ 7.2 ± 1 vs. 15 ± 4

^#^ Age shown as mean ± SD or range * *p* < 0.05 vs. control. ◊ By the 2007 ASE/EACVI [67] guidelines. LA, left atrium; HCM, hypertrophic cardiomyopathy; HFpEF, heart failure with preserved ejection fraction; AS, aortic valve stenosis; T, Telsa; TR, temporal resolution; ST, slice thickness, SR; spatial resolution.

## Data Availability

Not applicable.
